# Parental burnout and resilience intervention among Chinese parents during the COVID-19 pandemic

**DOI:** 10.3389/fpsyg.2022.1034520

**Published:** 2022-12-01

**Authors:** Yixiao Liu, Jing Han Chee, Ying Wang

**Affiliations:** ^1^Department of Psychology, Tsinghua University, Beijing, China; ^2^Department of Gastroenterology, State Key Laboratory of Complex Severe and Rare Diseases, Peking Union Medical College Hospital (PUMCH), Chinese Academy of Medical Sciences (CAMS), Peking Union Medical College (PUMC), Beijing, China

**Keywords:** child maltreatment, exercise training, meditation training, protective factor, risk factor

## Abstract

**Introduction:**

Parental burnout is a mental state that combines long-term stress and depression with an overwhelming feeling of parental pressure.

**Methods:**

In Study 1, we conducted a web-based survey of 390 Chinese parents (75.1% mothers) with children aged 1–18 years old (Mean age = 9.05 years, SD = 5.098) to examine the parental burnout during the COVID-19 global pandemic and to identify associated factors during the national lockdown. In Study 2, eight weeks of resilience intervention was administered to 20 parents to compare parental resilience and parental burnout before and after the intervention.

**Results:**

The correlational study showed that greater parental burnout occurred in parents with the lower educational levels and in parents of school-age children. The risk factors of parental burnout included household burden and children’s interference with work; while protective factors included living materials, family atmosphere, and parent–child meeting frequency. The intervention study showed the effectiveness of meditation intervention in resilience and parental burnout, suggesting that meditation training can effectively increase parental resilience and reduce parental burnout.

**Discussion:**

These findings demonstrate the risk and protective factors associated with parental burnout during the COVID-19 lockdown and highlight the positive role of meditation in mitigating parental burnout.

## Introduction

Tremendous levels of stress worldwide caused by the COVID-19 pandemic have greatly affected the mental health of the general population (Mazza et al., 2020). Parents who have children who are required to stay home from school have undoubtedly suffered due to the challenges associated with the lockdown: balancing family tasks and professional performance. This leads to serious parental burnout and, sadly, child violence, and neglect (Griffith, 2020; Han and Yang, 2020). Since child abuse and neglect cause negative effects that can last throughout a lifetime, it is critical for researchers, parents, and policymakers to recognize the causes and preventative strategies for parental burnout during the global pandemic. In China, with the outbreak of the COVID-19 epidemic occurring over the 2020 winter vacation season, all kindergartens, primary, and secondary schools postponed reopening, requiring students to live and study at home (China, 2020). According to an online survey of 10,000 participants, 44.9% of Chinese parents reported feeling stress, burnout, and depression during the pandemic (BNU, 2020). During lockdown, Chinese parents had to manage all family responsibilities while carrying out professional responsibilities at home. External sources of support for childcare or education were limited or nonexistent. This study focuses on the risk and protective factors associated with COVID-19-related burnout amongst Chinese parents, and further explores how resilience interventions can buffer this phenomenon and ultimately, promote the future recovery of severe worldwide events. Results in these topics present possibilities for improving mental health services aimed specifically at parents and families affected by the COVID-19 lockdown and for enhancing prevention strategies in the case of future public-health crises.

Parental burnout, defined as a prolonged response to chronic and overwhelming parental stress (Mikolajczak et al., 2019), consists of four main symptoms: physical and mental exhaustion, alienation from children, decline of parenting pleasure, and loss of parental responsibility and achievement (Mikolajczak et al., 2017). Some parents also report a trapped sensation from which escape is difficult (Hubert and Aujoulat, 2018). According to the Balance between Risks and Resources (BR^2^) theory, established by Mikolajczak and Roskam (2018), the imbalance between demands and resources within the parental role leads to the emergence of parental burnout syndrome. Thus, parents who have more demands placed on them but fewer mental and material resources to meet those demands are easily exhausted and susceptible to burnout. Conversely, once the resources exceed the demands, the occurrence of parental burnout decreases greatly. Additionally, environmental factors that interfere with normal family life, such as working part-time, staying home more, and having a disabled child, are regarded as risk factors for parental burnout (Gérain and Zech, 2019; Sorkkila and Aunola, 2020). Dispositional factors, like neuroticism, rigor, and self-oriented, socially-prescribed perfectionism, increase parental burnout, while other dispositional factors, like agreeableness and emotional stability, help mitigate it (Le Vigouroux et al., 2017; Mikolajczak et al., 2017; Sorkkila and Aunola, 2020). Furthermore, family functioning, such as marital satisfaction, the co-parenting situation, and family disorganization, are significantly correlated with parental burnout (Mikolajczak and Roskam, 2018). For instance, poor co-parenting significantly increased parental stress and predicted parental burnout (Mikolajczak and Roskam, 2018). Finally, practical and emotional support from others can also play protective roles and decrease levels of parental burnout (Lindstrom et al., 2011).

### Risks and resources of parental burnout

A recent study showed that family patterns changed significantly across OECD countries during the COVID-19 pandemic, and many of risk factors that contribute to parental burnout are more likely to happen during periods of national lockdown, including dramatic increases in household chores, job loss, working from home and household tension (Biroli et al., 2021). Due to many countries’ policies of working from home and maintaining social distancing, parents received less practical and emotional support, which also influenced the level of parental burnout (Brown and Shenker, 2021). However, most research has addressed the risks and protective factors of parental burnout in families of Western or Euro-American cultures and one recent across-countries study indicated that individualistic cultures may display a higher prevalence and mean level of parental burnout (Roskam et al., 2021). Thus, it is essential to explore the specific risk factors of parental burnout in different cultures, especially in collectivistic cultures and the negative effects of parental burnout on children during the COVID-19 pandemic. Results in this direction can help us understand the resources and coping strategies that enable them to prevent parental burnout and reduce the associated negative consequences for children in different cultures.

Recently some studies identified some unique risk and protective factors of parental burnout during COVID-19 in Eastern cultures. Zhang et al. (2022) found that Chinese parents who need to balance work and parenting tasks in the shared environment during lockdowns suffered from higher levels of parental stress. A Vietnamese study showed that students’ behavior problems are also a risk factor for parental burnout and will reduce children’s academic outcomes (Hong et al., 2022). Studies also demonstrated that parental perfectionism is one of the vulnerability factors of parental burnout in Japan (Kawamoto et al., 2018; Furutani et al., 2020). Another thing to be noted, in Eastern culture, children’s academic performance is of great concern to parents, and children are more likely to interpret their parents’ coercive tactics as evidence that they are loved. For these reasons, risk factors found in the Western country may not have the same effect on parents or children as it has in Eastern countries. Minh et al. (2022) found that parental psychological control and parental burnout played a serial mediating role in the relationship between children’s behavior problems and parents’ psychological well-being among Vietnamese parents. For the protective factors, previous studies revealed that cooperation and friendliness personality of parents and children, emotional regulation skills of parents and children’s independence are found as important protective factors of parental burnout in different cultures (Le Vigouroux et al., 2017; Vigouroux and Scola, 2018; Swit and Breen, 2022). Therefore, it is necessary to examine both the risk and protective factors of parental burnout in different Eastern cultures and the possible adverse effects of parental burnout on children. Since China relaxed its one-child policy in November 2013, and the couples are now allowed to have up to three children, which may cause higher parental burnout than before since the number of children is recognized as one of the risk factors in the previous studies (Mikolajczak and Roskam, 2018). Thus, investigating the characteristics of parental burnout in Chinese society during the COVID-19 pandemic is important, not only for ascertaining possible risk and protective factors associated with parental burnout, but also for suggesting resources and strategies for buffering the negative effects.

### The consequences of parental burnout

Another important question is if parental burnout has negative consequences on children’s development. The few studies that attempted to answer this question revealed that parental burnout negatively impacted both the parents burdened with high levels of parenting pressure and the children raised by these parents (Krabbe et al., 2017). For parents, while Mikolajczak et al. (2019) demonstrated that parental burnout strongly and linearly increased parents’ suicidal thoughts and escape ideation, and Ahola et al. (2006) noted the association of burnout with parents’ alcoholism and severe sleep problems. Long-term continuity in these unhealthy thoughts and behaviors leads to the deterioration of the family environment and causes irreversible damage to children’s development. Research revealed that parents who experience burnout were more likely to engage in higher levels of child abuse and other neglectful behaviors (Krabbe et al., 2017). As the frequency of these neglectful behaviors increased, parents were likely to experience more parental burnout (Holden and Banez, 1996), leading to a cycle of neglect and burnout. Yang et al. (2021) longitudinal study found that greater parental burnout was predictive of youth’s greater depressive and anxiety symptoms 2 months later, and such effects were partially mediated by less autonomy-supportive parenting. Chen B. B. et al. (2022) also found that mothers’ parental burnout can predict adolescents’ perceptions of their mothers’ parental hostility over time, which were in turn related to adolescents’ later internalizing and externalizing problems. Because of the acutely adverse effects that child abuse and neglect can have on an individual’s development across the lifespan, it is also necessary to examine the prevention measures of parental burnout during the global pandemic, in order to provide appropriate, evidence-based intervention strategies for governments and professional organizations to prevent these negative outcomes.

### Resilience intervention and parental burnout

The public health literature provides robust evidence on how interventions can mitigate negative outcomes in the aftermath of a global public disaster (Bauerle et al., 2020; Manucci, 2021). Previous studies have shown that cultivating and sustaining resilience is a preventive strategy for employment-related psychological burnout (Shin et al., 2012; Harker et al., 2016). A recent study of Chinese mothers’ parental burnout showed similar findings that compared with parents with lower resilience, parents with higher resilience perceived divergent social support and lower level of parenting burnout (Chen M. L. et al., 2022). In general, resilience is considered as an important psychological factor that leads to relatively stable personality characteristics and enables individuals to adapt to various encounters (Fletcher and Sarkar, 2013; Sarkar and Fletcher, 2014). Although resilience has been studied in multiple ways (Jacelon, 1997), researchers have found that resilience is not a stable but rather fluctuates as a function of life circumstance, social support and type of interventions (e.g., Migerode et al., 2012; Bajaj and Pande, 2016; Trigueros et al., 2020). In our study, we tested if meditation and exercise interventions can act as a buffer between the COVID-19 pandemic and parental burnout, through increasing resilience.

Meditation and exercise interventions can also significantly improve resilience and burnout in the work environment (Goldhagen et al., 2015). Bajaj and Pande’s (2016) intervention study showed that meditation improved an individual’s resilience by partially regulating their perceived life satisfaction (Bajaj and Pande, 2016). Several further studies demonstrated a relationship between exercise intervention and resilience, showing, for instance, that after a period of continuous exercise, an individual’s resilience level and mental health significantly improved (Belcher et al., 2021). In general, meditation and exercise have been shown to increase resilience levels in individuals and be effective in helping individuals cope with burnout situations. However, the relationship between resilience and parental burnout has yet to be investigated. The present study aimed to use physical exercise and meditation to intervene with levels of parental burnout and resilience.

### Research framework and hypothesis

In summary, the current study investigated the family and individual factors that may be associated with parental burnout during the COVID-19 outbreak in China. We were also particularly interested in understanding the negative effects of parental burnout and whether resilience intervention programs could reduce levels of parental burnout during the lockdown. Study 1 explored the correlates and consequences of parental burnout. In Study 2 we implemented the interventions to investigate the potential buffering effects of resilience on the relationship between the pandemic and parental burnout.

In the first study, we used online surveys to explore factors influencing parental burnout and burnout’s potential effects on child abuse and neglectful behaviors. We hypothesized that socio-demographic and environmental factors (i.e., socioeconomic status, having a school-age child, academic expectations, housework burdens, and family atmosphere) may significantly increase levels of parental burnout in Chinese society during lockdown, which may lead to higher levels of child abuse and neglectful behaviors.

## Study 1: A correlational study of parental burnout

### Method

#### Participants

A total of 390 participants (female = 293) who had at least one child aged 1 to 18 years old and stay at home were recruited for this study. All participants in the study completed an online questionnaire in November 2020. Since China had implemented strict epidemic management and lockdown policies starting in January 2020, participants in this study were all affected by nationwide control policies and experienced a long period of staying home with their families. All participants were parents of primary, secondary and kindergarten students in China. Of the participants, 172 (44.1%) had one child living at home, 192 (49.2%) had two, and 26 (6.7%) had three. The mean age of the youngest child in the participants’ families was 9.05 years (*SD* = 5.10 years). All participants were recruited from one of three northern cities in China and were from working-and middle-class backgrounds. With regard to parents’ educational level, 123 participants (31.5%) graduated from junior high school (compulsory education in China), 64 (16.4%) graduated from senior high school, 67 (17.2%) graduated from a higher vocational college, 115 (29.5%) earned a bachelor’s degree, and 21 (5.4%) earned a master’s or doctoral degree. Parents who participated in the study were the primary caregivers of their child/children (70% mothers; Mean age = 41.80 years, *SD* = 3.81 years). As for family background, 371 participants (95.1%) were married, 17 (4.4%) were divorced, and 2 (0.5%) were widowed.

#### Measurements

##### Parental burnout

The most widely used scale to accurately measure parental burnout is the Parental Burnout Assessment (PBA), which is based on the aforementioned theory of BR^2^ (Roskam et al., 2018). In this study, parental burnout was assessed using the Chinese version of the PBA, which was validated and shown to be reliable by Roskam et al. (2018) and Cheng et al. (2020). This version contained 21 items (e.g., “I have zero energy for looking after my child (ren)”), with each item assessed using a seven-point Likert scale (1 = *never*, 7 = *every day*). The sum of the scores across all items represented the level of parental burnout, with a higher score indicating greater parental burnout (*α* = 0.97).

##### Family status during COVID-19 pandemic

Family status was assessed using an adaptation of the structured online questionnaire developed by Abuzerr et al. (2021). Eight questions gathered information about family living conditions (e.g., household burden, living materials) and parental conditions (e.g., academic expectations, health concerns, family atmosphere, parent–child meeting frequency) during the pandemic. This questionnaire contained 8 items and each item was assessed using an 11 point scale (e.g., “I was very worried that my child (ren)'s education would be affected during the pandemic” for-5 and “I was not worried at all that my child (ren)'s education would be affected during the pandemic” for +5). The sum of the scores across all items represented the family status, with a higher score indicating more positive family status (*α* = 0.78).

##### Parental neglect

Parental neglect was assessed using the Parental Neglect Scale (PNS), designed by Mikolajczak et al. (2019). The scale contained three items corresponding to the three aspects of neglect: physical, educational, and emotional neglect. The sum of the items corresponded to the level of parental neglect (*α* = 0.82).

##### Parental violence

Parental violence was assessed using the Parental Violence Scale (PVS), designed by Mikolajczak et al. (2019). The scale contained three items corresponding to the three aspects of violence: verbal, physical, and psychological violence. The sum of the items corresponded to the level of parental violence (*α* = 0.74).

For the questionnaires of Family status during COVID-19 pandemic, parental neglect and parental violence, forward-and back-translations were performed to confirm accuracy. The forward-translation was done by this paper’s co-first authors. Translations were reviewed and discussed with the corresponding author and another bilingual Ph.D. student. A revised version was back-translated by two bilingual Ph.D. students at Tsinghua University. All individuals involved in the translation are fluent in English and Chinese and majored in psychology. These questionnaires were translated into Chinese and tested for wording acceptability. The original and back-translated English versions were compared, and inconsistencies were resolved through consensus meetings. The Chinese version was finalized when there was no dispute or new suggestions.

#### Procedures

Online questionnaire was used in current study. We collected a total of 443 questionnaires from 390 parents who matched our eligibility criteria. These criteria included having at least one child younger than 18, responding to the questionnaire for at least 10 min, passing the attention check (including 3 instructed response items in the questionnaire, such as “Please indicate option [always] for this question”), and not exhibiting apparent contradictions across questions. The online survey focused on five categories of factors: sociodemographic scale, parental burnout, family status during the pandemic, parental neglect and violence, and level of resilience. After completing the survey, participants could choose to leave contact information to participate in follow-up resilience intervention programs. The study was approved by the university Ethics Committee. Participants completed out an online consent form before completing the survey and taking part in the intervention study.

#### Data analyses

Firstly, we performed descriptive statistics for all the variables measured, including ranges, means, standard deviations, skewness and kurtosis. Next, we explored the effects of different demographic variables on parental burnout, independent samples t-tests were applied to test binary variables and Pearson’s correlation test were applied to test multiple categorical variables. Demographic variables that have a significant effect on parental burnout would be controlled for the next step. To explore the possible risks and protective factors in family status that influenced parental burnout, we entered all items from the COVID-19 family status questionnaire into a partial correlation model while controlling the significant demographic variables shown in the previous step. In order to further explain the variance in parental burnout, we conducted a hierarchical regression analysis with the variables that were significantly associated with parental burnout. Finally, we examined the consequences of parental burnout by analyzing correlations between the levels of parental burnout and parental violence and neglectful behaviors at home.

For all the analyses, we used SPSS version 25.0.

### Results

The ranges, means, standard deviations, skewness and kurtosis for parental burnout, neglect, violence, and resilience are displayed in Table 1. Firstly, we examined the relationships between demographic factors and parental burnout. Since over 99.5% of the participants were married or divorced, while calculating correlation, marital status was considered a binary variable and the widowed participants (0.5% of all participants) were grouped with the “married.” We analyzed the relationships between gender, marital status and parental burnout using independent samples t-test. Our results showed that there were no significant relationships between gender (*t* = 0.334; *p* = 0.738) or marital status (*t* = 0.006; *p* = 0.995) and level of parental burnout. Since levels of parental burnout showed no difference between parents’ gender or marital status, we did not control these two variables in the subsequent analyses.

**Table 1:** Ranges, means, and standard deviations for all variables (*N* = 390).

Variable	Range	*M*	*SD*	Skewness	Kurtosis
Academic expectation	1–11	5.60	3.64	0.15	−1.41
Trust in distance education	1–11	5.81	3.34	0.00	−1.21
Household burden	1–11	8.48	2.79	−1.01	0.14
Living materials	1–11	8.52	2.68	−1.08	0.49
Health concern	1–11	5.98	3.52	0.00	−1.38
Children’s interference with work	1–11	8.55	2.63	−0.96	0.13
Family atmosphere	1–11	8.66	2.47	−0.90	−0.05
Parents-children meeting frequency	1–11	9.57	2.19	−1.70	2.43
Parental burnout	21–147	43.25	28.27	1.64	2.03
Parental neglect	2–23	8.40	6.95	0.86	−0.56
Parental violence	2–23	4.88	3.95	2.25	5.71
Resilience	60–180	133.59	18.54	0.043	1.14

Table 2 shows all correlations among other included variables. Parents’ education level (*r = −0*.134; *p* = 0.008) and age of the youngest child (*r = 0.*110; *p* = 0.030) were significantly associated with parental burnout. Resilience was significantly associated with monthly income (*r* = 0.134; *p* = 0.008). The results demonstrated that a higher educational level was associated with a lower degree of parental burnout, while having a youngest child in the family that was older in age was associated with a higher degree of parental burnout. We further divided the youngest child into two groups, pre-school (0–6 years old) and school-age (7–18 years old), and explored the impact of group differences in parental burnout. The results of an independent sample t-test showed significant group differences in parental burnout (*t* = −2.705, *p* = 0.007), with parents raising school-age children (*M* = 46.89; *SD* = 32.36) having significantly higher levels of parental burnout than those raising pre-school-aged children (*M* = 42.32; *SD* = 27.09). The result of partial correlation model while controlling for education level and age of the youngest child showed that household burden (*r* = 0.23, *p* < 0.001), living materials (*r* = −0.23, *p* < 0.001), children’s interference with work (*r* = 0.23, *p* < 0.001), family atmosphere (*r* = −0.36, *p* < 0.001), and parent–child meeting frequency (*r* = −0.28, *p* < 0.001) were significantly correlated with parental burnout. These significant correlations allowed us to classify household burden and children’s interference with work as significant risk factors of parental burnout, while living materials, family atmosphere, and parent–child meeting frequency as protective factors of parental burnout.

**Table 2:** Correlations among variables (*N* = 390).

Variable	1	2	3	4	5	6
1. Education level						
2. Monthly income	0.542**					
3. Number of children	−0.321**	−0.211**				
4. Age of youngest child	−0.305**	−0.206**	0.001			
5. Family number	0.148**	0.211**	0.324**	−0.369**		
6. PBA	−0.134**	−0.091	0.033	0.110*	−0.081	
7. Resilience	−0.003	0.134**	0.027	−0.091	0.084	−0.114*

*p < 0.05; **p < 0.01.

In the hierarchical regression analysis, educational level and age of the youngest child were entered in Block 1; risk factors (household burden and children’s interference with work) were entered in Block 2; and protective factors (living materials, family atmosphere, and parent–child meeting frequency) were entered in Block 3. As indicated in Table 3, with education level and age of youngest child statistically controlled, risk factors explained 7.3% unique variance and protective factors explained 8.0% unique variance in levels of parental burnout. Thus, the regression coefficients of household burden (β = 0.167, *p* = 0.002), children’s interference with work (β = 0.164, *p* = 0.002), family atmosphere (β = −0.258, *p* < 0.001), and parent–child meeting frequency (β = −0.154, *p* = 0.003) all emerged as unique correlates of parental burnout.

**Table 3:** Linear regression analysis: Best model to predict parental burnout.

Block and variable	*R* ^2^	Δ*R*^2^	*B*	*SE*	β
Block 1					
Education level	0.023	0.023*	−2.378	1.117	−0.112*
Age of the youngest child			0.412	0.293	0.074
Block 2					
Household burden	0.096	0.073**	1.697	0.532	0.167**
Children’s interference with work			1.758	0.561	0.164**
Block 3					
Living materials	0.177	0.080**	−0.135	0.805	−0.013
Family atmosphere			−2.957	0.676	−0.258**
Parents-children meeting frequency			−1.995	0.674	−0.154**

*p < 0.05; **p < 0.01.

After controlling for educational level of parents and age of the youngest child, results showed significant positive correlations between parental burnout and violent behaviors (*r = 0.*708; *p < 0.*001) and between parental burnout and neglectful behaviors (*r = 0.*404, *p < 0.*001).

### Discussion

The present study focused on the predictors and consequences of parental burnout in Chinese society during the COVID-19 pandemic. Our results confirmed our hypothesis suggesting that sociodemographic factors (parents’ education level and age of the youngest child) and family status (household burden, children’s interference with work, living materials, family atmosphere, and parent–child meeting frequency) significantly influenced parental burnout. Furthermore, results indicated a significant positive correlation between parental burnout and violent and neglectful behaviors, indicating that as levels of parental burnout increased, Chinese parents behaved more neglectfully and violently towards their children.

#### Risks and protective factors of parental burnout

Our results showed that educational level negatively correlated to parental burnout, while age of the youngest child positively correlated to it. That is, the higher the educational level and the younger the age of the youngest child, the less parents experienced burnout. Although some previous studies showed that most sociodemographic factors, including gender, age, educational level, and net income, barely influenced parental burnout (Le Vigouroux et al., 2017; Mikolajczak et al., 2017), under a more authoritarian and academically-oriented environment like many Chinese households, parents’ educational level and age of the youngest child emerged as significant predictors of parental burnout. Previous studies also found that parental education level was a significant factor influencing parental burnout in the context of pandemic lockdown, and higher education levels led to a higher level of well-being, positive affect, and reduced psychological distress (Aguiar et al., 2021; Tran et al., 2021). Parents with higher education level might be more open-minded to different viewpoints and lifestyles related to positive well-being, whereas parents with lower education level might be more likely to experience stress and find it more difficult to adjust to multiple roles in the family and at work, since they may receive less social support from family, colleagues and social communities.

We also found that parents with older children, especially school-age children, experienced a higher level of parental burnout, compared to parents with preschoolers. Due to Dong et al. (2020)‘s study, the implementation of online learning during the pandemic has been problematic and challenging for Chinese families and Chinese parents were neither trained nor ready to embrace online learning. Therefore, this unusual phenomenon may be explained by the emphasis Chinese parents place on their children’s learning, as well as on parents’ strict disciplinary style and expectation for their children to attend online classes. Parents with school-age children experience greater pressure and have higher expectations for their children’s academic progress: in a survey of 19,487 Chinese middle school students, more than 30% reported that their parents expected them to reach the top five of their class, and almost 90% were expected to earn a college degree or higher (Li et al., 2017). Another study focused on middle-class families in China also confirmed that Chinese parents place a high expectation on their children (Zou et al., 2013). These high expectations, coupled with stricter disciplinary style, may be the driver of this phenomenon.

In regard to family status during lockdown, two risk factors of parental burnout—household burden and children interfering with work—and three protective factors of parental burnout—living materials, family atmosphere, and parents-children meeting frequency—were identified. Household burden has long been recognized as a cause of stress and burnout, especially in East Asian cultures. For example, researchers in both Japan and Korea reported that housework was an important source of family stress that could lead to severe psychological disorders, including depression and suicide (Hoshino et al., 2016; Lee et al., 2018). During the pandemic, household burden became a significant source of parental burnout because parents were home for long periods of time with more family members. Children interfering with work and living materials were two other factors that developed during the lockdowns, since in pre-pandemic times, children had attended school during the day, and parents neither needed to work from home nor stock up on large quantities of large quantities of food and daily essentials. The combination of increased household burden, more interference between children and work, and a lack of living materials during the COVID-19 pandemic created an especially difficult and stressful living situation that greatly amplified the level of parental burnout. Family emotional atmosphere has been found to significantly affect nurses’ emotional distress tolerance in the context of the COVID-19 pandemic (Ensafdaran et al., 2022). Combined with research results, family atmosphere and parent–child meeting frequency were protective factors, suggesting the importance of creating a warm and comfortable family atmosphere, reducing family disputes, and increasing parent–child interaction in daily life.

#### Negative effects of parental burnout on abusive and neglectful behaviors

Our results showed that parental burnout correlated to a higher frequency of violent behaviors, which is in line with previous findings (Clément and Chamberland, 2008; Hansotte et al., 2020). Violence may be seen as a way for parents to release their pent-up stress (Pereda and Díaz-Faes, 2020). Chinese parents may also experience higher levels of this stress, when supervising their children’s online schooling and homework, and thus, be more likely to use violence against their children (Mikolajczak et al., 2018). Approaching from the children’s side, children’s oppositional behaviors may increase due to the lockdown, eliciting harsher responses and more violent behaviors from parents (Humphreys et al., 2020).

We also found that parental burnout also led to an increase in neglectful behaviors. Though many theories exist to explain parents’ neglectful behaviors (Hildyard and Wolfe, 2002), a stressful situation (the COVID-19 pandemic and associated lock-down) was the main concern in our study. When parents encounter burnout, neglectful behaviors may signal their desire to escape from their roles as parents. Neglectful behaviors are also a type of self-protective mechanism that are automatically triggered in response to stressful situations and that parents may use in an effort to overcome their situations and regain personal control (Guterman et al., 2009).

Study 1 investigated the family and individual factors associated with parental burnout during the COVID-19 pandemic in Chinese society and the associated negative effect parental burnout has.

In Study 2, we investigated the protective role of resilience and tested an intervention program aimed toy reduce levels of parental burnout. We hypothesized that resilience is negatively correlated with parental burnout levels, and meditation and physical exercise interventions can decrease parental burnout levels through increasing resilience levels.

## Study 2: The effects of resilience interventions on parental burnout

### Method

#### Participants

In the resilience intervention, we recruited 30 participants who were matched by socioeconomic status (monthly income ranged from 10,000 RMB to 30,000 RMB) from Study 1, and randomly separated them into three groups: the meditation group, the exercise group, and the control group. The number of female and male participants did not differ significantly between the three groups.

#### Measurements

##### Parental burnout

In this study, parental burnout was assessed using the same Chinese version of the Parental Burnout Assessment that was used in Study 1.

##### Resilience

Resilience was measured using a Chinese version (Wang et al., 2014) of the Connor-Davidson Resilience Scale (Connor and Davidson, 2003) and included 36 items, each rated on a 5-point scale (1 = completely disagree, 5 = completely agree). The sum of all items represented the level of resilience, with higher scores corresponding to greater resilience (α = 0.95).

#### Procedures

Participants completed an online questionnaire asking about the levels of parental burnout and resilience before and after the intervention. The questionnaire included the two measures mentioned above (the Parental Burnout Assessment and the Connor-Davidson Resilience Scale) and basic demographic information. We randomly divided participants into three groups, the meditation group, the exercise group and the control groups. The meditation and exercise groups aimed to promote parents’ resilience level and decrease their parental burnout level. Each week, participants in the intervention group finished a designated task, while participants in the control group did not need to complete any tasks. The entire intervention study lasted 8 weeks (from January 2021 to March 2021).

##### The meditation group

The meditation group participants enrolled in an online meditation training course through an app called “Meditation Planet.” Each meditation session ranged from 10 to 40 min once a week, for a total of 8 weeks. Topics of each training were different, including breathing exercises, mindfulness stretches, body scans, and other practices. The course materials were designed by the Center for Mindfulness at the University of Massachusetts School of Medicine. After every session, participants were required to upload a photographic or video record to the WeChat group used to monitor intervention participation.

##### The exercise group

The exercise group participants engaged in sessions of aerobic exercises (e.g., jogging, swimming) for at least 1 h each week, for a total of 8 weeks. Participants were able to choose whatever aerobic exercises they liked. Like participants in the meditation group, after every session, participants in the exercise group were required to upload a photographic or video record to a WeChat group that we used to monitor participation.

##### The control group

Participants in the control group were asked to complete the online questionnaire at the beginning and end of the intervention period for participants in the other two groups. Participants in the control group were provided the same opportunity to do the mediation and exercise at the end of the study.

#### Data analyses

For the intervention, we first examined changes in each group before and after the intervention using t-tests, and used the *post hoc* analysis to performed the comparison of parental burnout among three groups after the intervention. Secondly, we used the mediation analysis to examine the relationship among meditation intervention, parents’ resilience level and parental burnout level, with groups (the intervention group and the control group) as the independent variable, the variation of parental burnout (post-intervention burnout level minus pre-intervention burnout level) as the dependent variable, and the variation of parents’ resilience as the mediator (post-intervention resilience level minus pre-intervention resilience level). All of the data analysis was done by SPSS version 25.0.

### Results

In this intervention study, we assessed parents’ resilience and parental burnout levels before and after the intervention. After controlling for parents’ education level, the age of the youngest child, and the monthly income of the family, partial correlation analysis showed a significant negative correlation between parental burnout and resilience before the intervention (*r* = −0.108, *p* = 0.034). We have described further findings below, by group.

#### The meditation group

The 10 participants in the meditation group showed significantly higher resilience scores after receiving the intervention (M_resilience_ = 146.00, *SD* = 15.36), compared to before (M_resilience_ = 114.70, *SD* = 22.03), *t (9)* = −3.935, *p = 0*.003. Parental burnout scores (M_burnout_ = 58.20, *SD* = 34.56) significantly decreased after the intervention (M_burnout_ = 29.50, *SD* = 9.03), *t (9)* = 2.802, *p = 0*.021. These results indicated that meditation intervention is able to effectively decrease parental burnout while increasing resilience.

#### The exercise group

The 10 participants in the exercise group showed no significant difference in resilience scores after receiving the intervention (M_resilience_ = 133.20, SD = 15.38), compared to before (M_resilience_ = 139.30, *SD* = 17.83), *t (9)* = −0.605, *p = 0*.560. Their parental burnout scores (M_burnout_ = 34.10, *SD* = 24.42) were also not significantly different after the intervention (M_burnout_ = 28.90, *SD* = 7.51), *t (9)* = −1.029, *p = 0*.330. These results indicated that exercise intervention had no effect on either resilience or parental burnout.

#### The control group

The 10 participants in the control group showed significantly lower resilience scores after 8 weeks (M_resilience_ = 96.60, *SD* = 14.206), compared to before (M_resilience_ = 114.70, *SD* = 22.03), *t (9)* = 6.672, *p < 0.*001. Parental burnout scores (M_burnout_ = 35.00, SD = 18.26) significantly increased after 8 weeks (M_burnout_ = 85.2, *SD* = 8.84), *t (9)* = −8.069, *p < 0.*001. The control group demonstrated results that were the opposite of results of the meditation group, after the 8 weeks.

#### Group differences in resilience and parental burnout

After controlling the parental burnout levels before the intervention, we used *post hoc* analysis to compared parental burnout levels between the three groups after the intervention. We found both the meditation group and exercise group scored significantly lower than the control group in levels of parental burnout (ps < 0.001), but there was no significant difference between the meditation group and the exercise group (*p* = 0.877).

After controlling resilience scores before the intervention, we found both the meditation group and exercise group scored significantly higher than the control group in resilience (*p* = 0.013, *p* = 0.020), but there were no significant differences between the meditation group and the exercise group (*p* = 0.527).

#### Mediation effects analysis

After analysis the outcome of our intervention study, we hypothesized that parental burnout could be decreased by resilience intervention, Thus, we conducted a mediation analysis using the regression model in SPSS, with groups (the intervention group and the control group) as the independent variable, the variation of parental burnout as the dependent variable, and the variation of parents’ resilience as the mediator.

**Figure 1:**
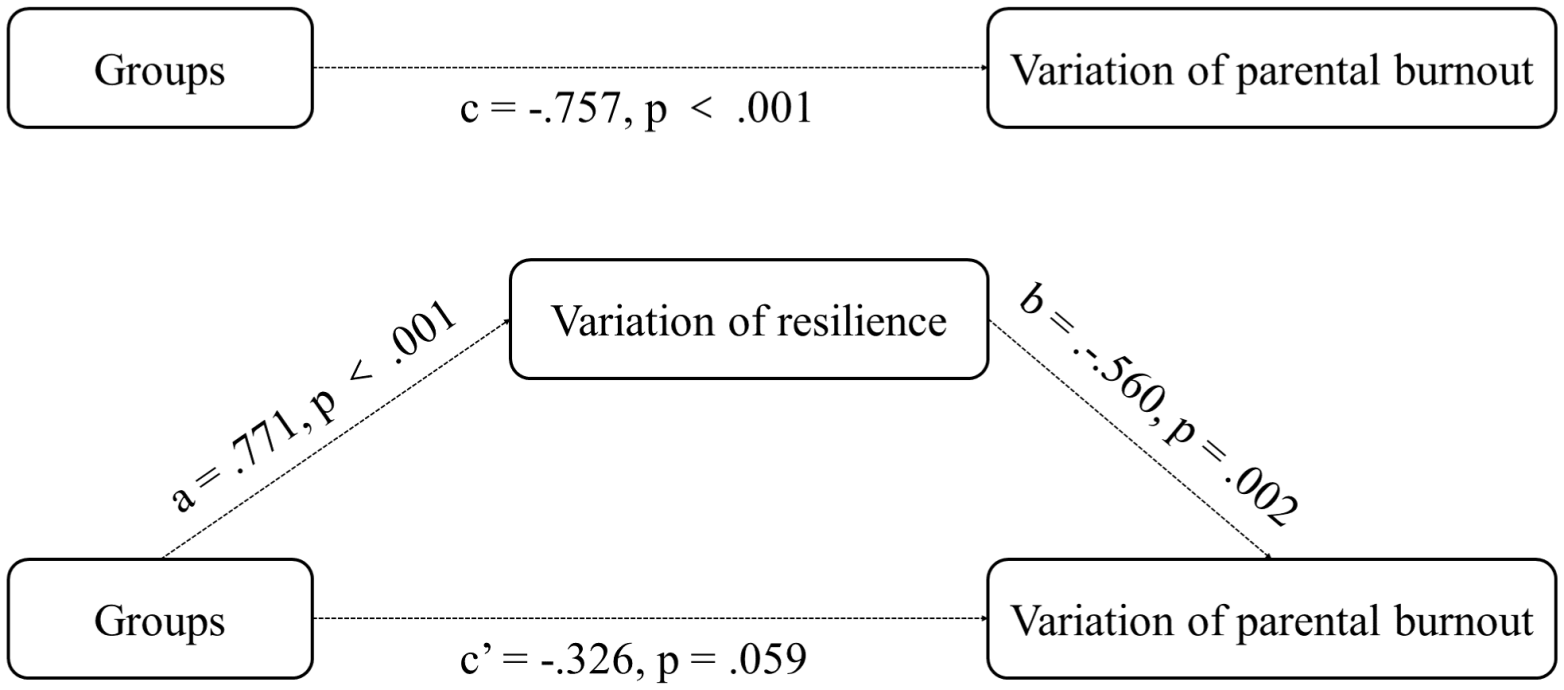
Simple mediation model evaluation the effect of the variation of resilience as a mediator between intervention and the variation of parental burnout, data is expressed by value analyzed by a linear regression model.

Results of the mediation analysis are shown in Figure 1. First, we found that intervention significantly predicted variation in parental burnout (*B* = −67.15, SE = 10.939, *b* = −0.757, t (1) = 6.139, *p* < 0.001). Second, intervention also significantly predicted the variation in parents’ resilience (*B* = 57.5, SE = 8.986, *b* = 0.771, t (1) = −6.399, *p* < 0.001). Third, variation in resilience predicted variation in parental burnout (*B* = −0.666, SE = 0.196, *b* = −0.560, t (27) = −3.394, *p* = 0.002). Finally, the direct effect of group and variation in parental burnout was not significant (*p* = 0.059). In conclusion, we found that variation in resilience played a complete mediating role in this model.

### Discussion

#### Benefits of resilience intervention for parental burnout

Our eight-week intervention study revealed that meditation significantly increased parents’ resilience while reducing parental burnout. Flynn et al. (2021) found that individuals with higher levels of resilience exhibited faster physiological and emotional recovery from negative consequences; parental burnout can be seen as a negative consequence parents face. Previous studies showed that meditation effectively benefitted self-control and development (Brown and Ryan, 2003; Kiken and Shook, 2012), which can help parents better cope with parenting stress during the pandemic (Behan, 2020). Researchers also found that meditation played a protective role against burnout and stress (Shapiro et al., 2012) and enhanced emotional regulation, by decreasing behavioral responses to acute psycho-social stressors (Basso et al., 2019). Thus, meditation can effectively help parents increase resilience and decrease parental burnout.

Although we did not find significant improvements in parents’ levels of resilience and parental burnout in the exercise group before and after the intervention, exercise may still act as a buffer against parents’ mental health problems, including burnout. We offer two discussions explanations for our findings. First, since the intervention lasted for only 8 weeks, this relatively short span of time might not have been enough time to show significant changes. Most studies that have demonstrated the benefits of exercise on improving resilience level utilized a program that lasted for more than 3 months (Hovell et al., 2008; Kettunen et al., 2015). Second, our results indicated that parents in the control group showed a higher burnout level and a lower resilience level at the second time point of data collection, meaning that after 2 months of staying at home, parents who received no intervention had a higher burnout level than exercise group participants, who demonstrated no significant changes in burnout levels. In other words, even though parents in the exercise group failed to show significantly higher resilience levels and lower burnout levels, it is noteworthy that their burnout levels did not increase over time.

Previous studies have found the protective role of meditation and exercise interventions on individual’s resilience (e.g., Goldhagen et al., 2015; Kim and Kang, 2017). In our study, results showed the significant mediating role of resilience, suggesting that the reduction in parental burnout levels was achieved by raising the participants’ resilience levels through meditation intervention. The mediating effect of parents’ resilience emphasize that mediation intervention could directly and significantly improve parents’ resilience level, which further buffer parental burnout level. Sorkkila and Aunola (2022) study supported our findings that they investigated 1,105 Finnish parents, and found that resilience predicted parental burnout negatively even after controlling other correlated variables.

## Limitation and general discussion

As an empirical study on the relationship between parental burnout and resilience in the context of the epidemic in China, this study still has some limitations. First, because of the lockdown, we collected data from internet, although there is more and more online research (e.g., Mikolajczak et al., 2018; Aguiar et al., 2021; Sorkkila and Aunola, 2022), the sampling of subjects may not take into account all groups. Second, the sample size of intervention study was rather small, the effect of the intervention need to be further confirmed in the future studies with more representative sample. Third, the resilience intervention only lasted 8 weeks and we only found the immediate effects of meditation intervention on resilience and parental burnout, in the future study we aim to conduct a long-term resilience intervention and examine both immediate and lasting effects.

Nevertheless, our findings determined the risk and protective factors influencing parental burnout, demonstrated its negative effects, identified an effective intervention for mitigating parental burnout, and emphasized the protective role of resilience in parental burnout during the pandemic-induced lockdown in China.

Globally, mental health has been one of the most challenging problems that have arisen during the COVID-19 pandemic. While there are uncertainties about how the pandemic will influence parents, children, and family functioning in the long-term, what is clear is its significant impact on parents’ and children’s mental health and psychosocial well-being. Our study found a dramatic increase in both pandemic-related parental burnout levels, child abuse, and other neglectful behaviors in Chinese society. Both a UNICEF report (UNICEF, 2021) and a recent review of current research (Krause et al., 2021) highlight the urgent need for better prevention and treatment for family members experiencing mental health issues. Our study suggests that the government should pay more attention to less educated parents with school-aged children, since these parents are more likely to experience serious parental burnout. Strengthening the supply of living materials and providing more educational support can effectively reduce their parental burnout levels, which, in turn, can help to decrease conflict between family members and increase the frequency of parent–child interaction. Finally, for those who are experiencing or have experienced high levels of parental burnout, meditation and exercise can relieve some parenting stress and enable parents to better interact with their children. Thus, communities and professional organizations can help to implement meditation and exercise intervention programs, in addition to psychological consultation and other interventions. The way researchers, clinicians, and communities work together now to tackle these mental health challenges will impact our collective drive for a successful global recovery and stronger societies in the future.

To the best of our knowledge, the current study was the first to explore the effect of resilience interventions on parental burnout in a Chinese context during the COVID-19 pandemic. Through a combination of web-based surveys and resilience intervention, we discovered and illustrated a possible method to reduce levels of parental burnout. However, this study has several limitations that must be considered. First, since data was collected at only two time points, this may not reflect the full scope of the situation. Moreover, although online surveys are a common method of scientific investigation during the pandemic (Wardropper et al., 2021), there was far less researcher oversight in the completion of our surveys, and thus, data could have been limited by the carelessness and/or impatience of participants. Second, we did not compare cultural differences of pandemic-induced parental burnout. Since parental burnout is strongly associated with different parenting styles and social systems across cultures, it is necessary to compare parental burnout between different cultural contexts and languages, in order to provide more prevention strategies targeted at a particular culture. Therefore, further longitudinal research in across countries and cultures during the ongoing COVID-19 pandemic is needed.

## Conclusion

In conclusion, findings from this study clearly demonstrated that both family and individual status can influence the levels of parental burnout in Chinese society during the COVID-19 pandemic. Meditation training can effectively increase parents’ resilience levels and reduce parental burnout. Our findings provide an optimistic outlook for parents in the on-going pandemic and in future lockdowns, as they may be able to manage their resilience and burnout levels through knowledge of risk factors, protective factors, and meditation.

## Data availability statement

The raw data supporting the conclusions of this article will be made available by the authors, without undue reservation.

## Ethics statement

The studies involving human participants were reviewed and approved by Department of Psychology Ethics Committee, Tsinghua University (DPEC). The patients/participants provided their written informed consent to participate in this study.

## Author contributions

YW supervised the study. YL and JC conducted the whole study, did the data collection, and performed the data analysis. All authors contributed to conception and design of the study, manuscript revision, read, and approved the submitted version.

## Funding

This study was supported by a grant from the National Natural Science Foundation of China (No. 31900771).

## Conflict of interest

The authors declare that the research was conducted in the absence of any commercial or financial relationships that could be construed as a potential conflict of interest.

## Publisher’s note

All claims expressed in this article are solely those of the authors and do not necessarily represent those of their affiliated organizations, or those of the publisher, the editors and the reviewers. Any product that may be evaluated in this article, or claim that may be made by its manufacturer, is not guaranteed or endorsed by the publisher.
